# Supplementary material for: Transcriptomic Insights into Mechanisms of Early Seed Maturation in the Garden Pea (Pisum sativum L.)
Source: Cells. 2020 Mar 23;9(3):779. doi: 10.3390/cells9030779 (PMC7140803; doi:10.3390/cells9030779)
Supplement: Supplementary file 1 [file cells-09-00779-s001.zip › Supplementary materials/FileS3.zip!/FileS2/Hormones - Results/Summary Sheets - Modules/K-Means Cluster/Report.pdf]

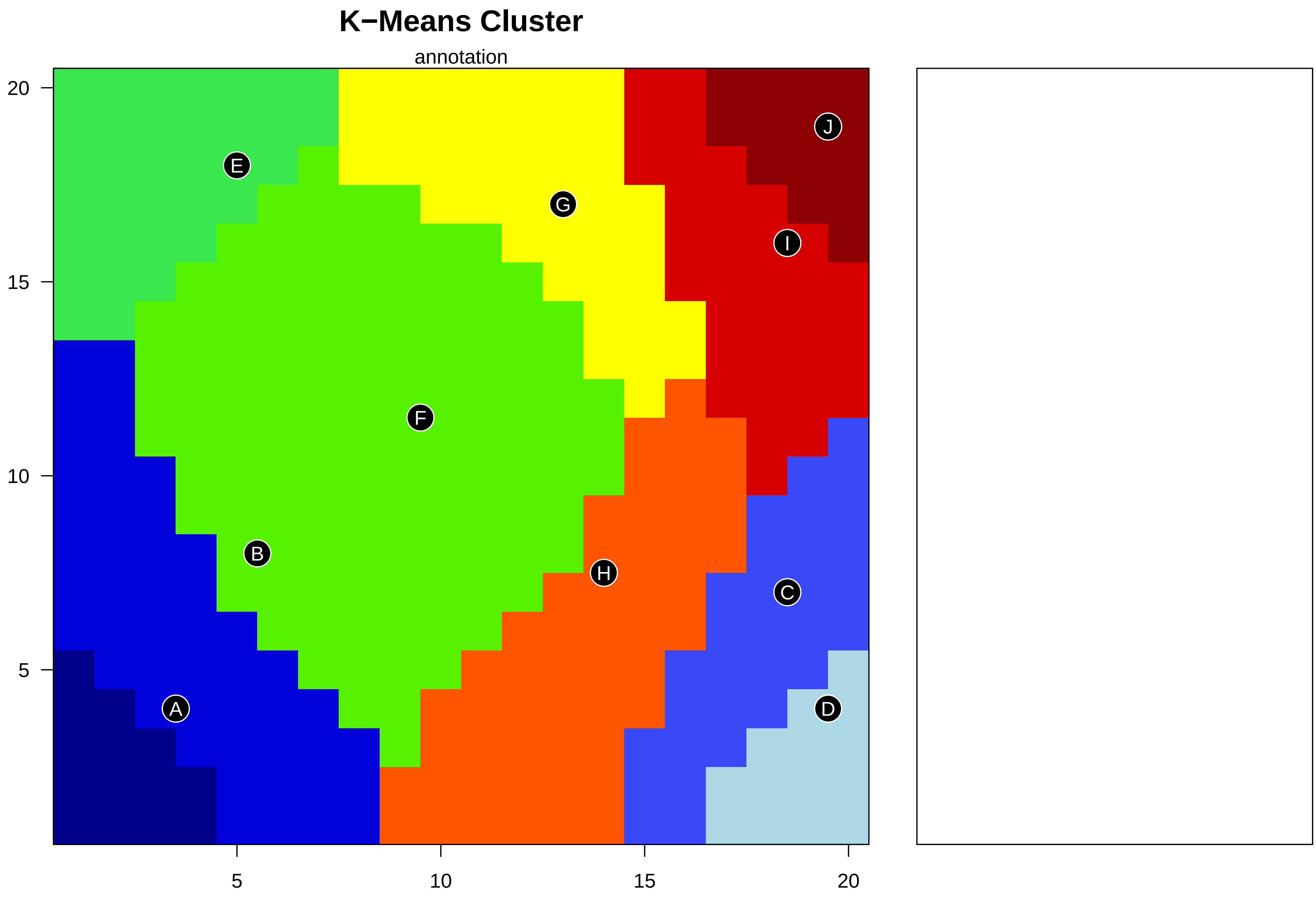
K−Means Cluster
annotation
20
15
10
5
5
10
15
20
A
B
C
D
E
F
G
H
I
J

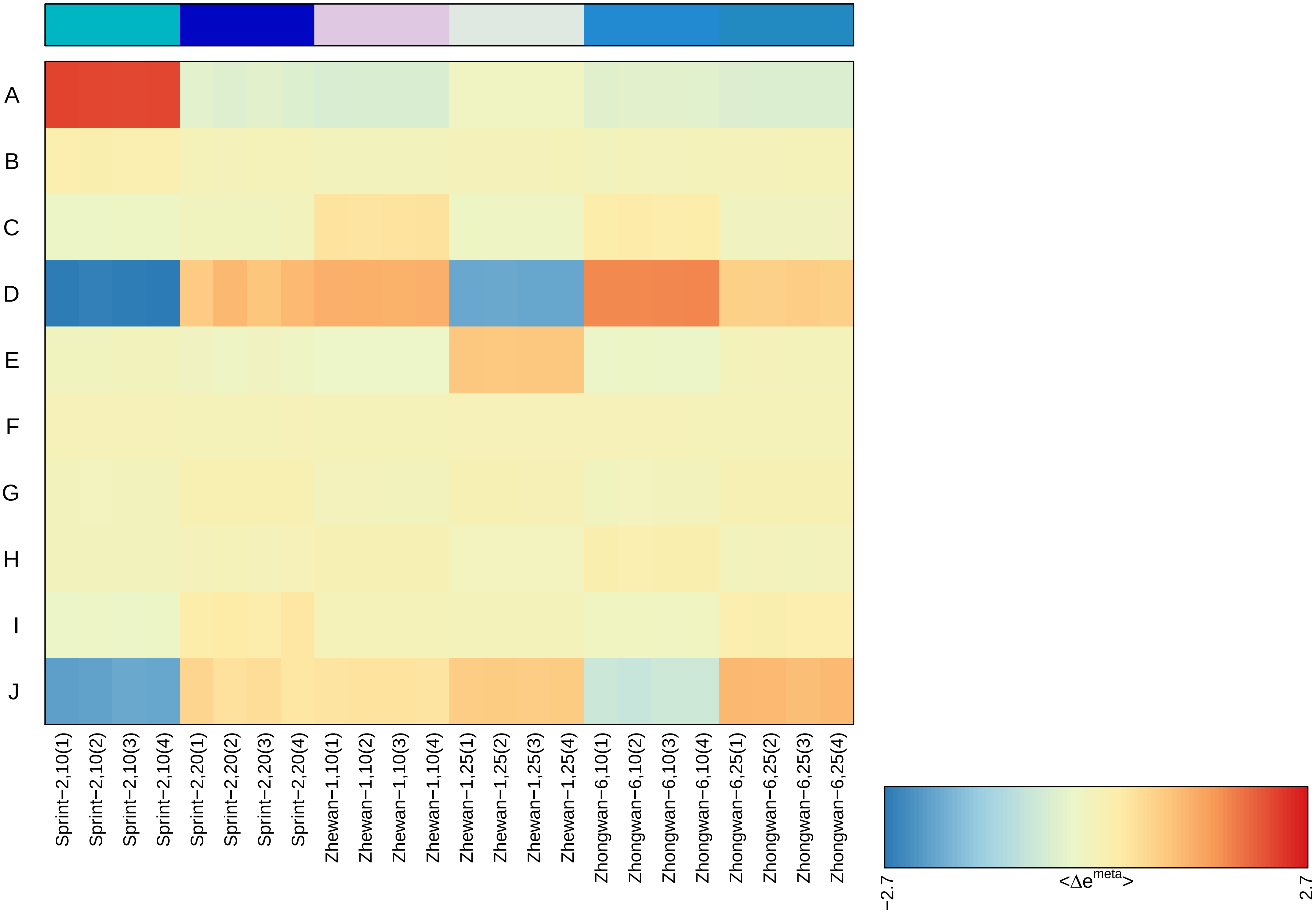
A
B
C
D
E
F
G
H
I
J
Sprint−2,10(1)
Sprint−2,10(2)
Sprint−2,10(3)
Sprint−2,10(4)
Sprint−2,20(1)
Sprint−2,20(2)
Sprint−2,20(3)
Sprint−2,20(4)
Zhewan−1,10(1)
Zhewan−1,10(2)
Zhewan−1,10(3)
Zhewan−1,10(4)
Zhewan−1,25(1)
Zhewan−1,25(2)
Zhewan−1,25(3)
Zhewan−1,25(4)
Zhongwan−6,10(1)
Zhongwan−6,10(2)
Zhongwan−6,10(3)
Zhongwan−6,10(4)
Zhongwan−6,25(1)
Zhongwan−6,25(2)
Zhongwan−6,25(3)
Zhongwan−6,25(4)
−2.7
<Δe^meta>
2.7

# K−Means Cluster

## Spot Summary: A

# metagenes = 14
# genes = 21

<r> metagenes = 1
<r> genes = 0.82
beta: r2= 1.73  /  log p= −Inf

# samples with spot = 4 ( 16.7 %)
Sprint−2(10) : 4 ( 100 %)

### Overview Map

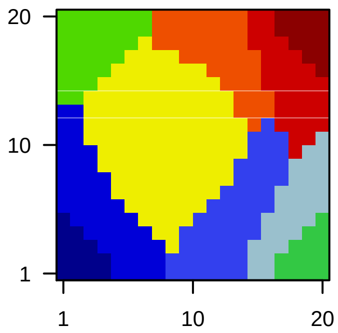


### Spot

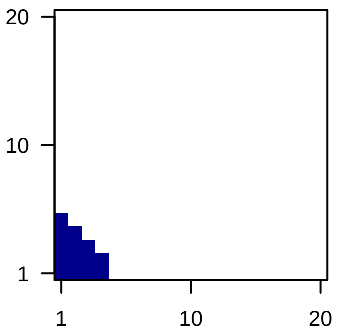


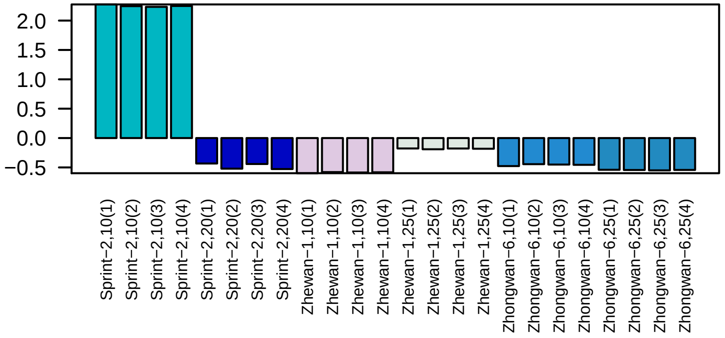


## Spot Genelist

| Rank | ID | max e | min e | r | Symbol | Description |
|---|---|---|---|---|---|---|
| 1 | TRINITY_DN1 | 15.48 | −3.34 | 0.91 | | |
| 2 | TRINITY_DN3 | 15.03 | −3.06 | 0.97 | | |
| 3 | TRINITY_DN4 | 4.64 | −1.14 | 1 | | |
| 4 | TRINITY_DN8 | 3.6 | −0.71 | 0.99 | | |
| 5 | TRINITY_DN4 | 3.56 | −0.75 | 0.98 | | |
| 6 | TRINITY_DN8 | 3.06 | −0.71 | 0.98 | | |
| 7 | TRINITY_DN6 | 2.31 | −0.98 | 0.36 | | |
| 8 | TRINITY_DN3 | 1.81 | −0.35 | 0.89 | | |
| 9 | TRINITY_DN3 | 1.66 | −0.44 | 0.96 | | |
| 10 | TRINITY_DN1 | 1.51 | −0.8 | 0.82 | | |
| 11 | TRINITY_DN5 | 1.42 | −0.47 | 0.86 | | |
| 12 | TRINITY_DN1 | 1 | −0.39 | 0.83 | | |
| 13 | TRINITY_DN2 | 0.93 | −0.39 | 0.97 | | |
| 14 | TRINITY_DN1 | 0.85 | −0.17 | 0.98 | | |
| 15 | TRINITY_DN2 | 0.69 | −0.19 | 0.97 | | |
| 16 | TRINITY_DN8 | 0.62 | −0.27 | 0.72 | | |
| 17 | TRINITY_DN1 | 0.54 | −0.21 | 0.96 | | |
| 18 | TRINITY_DN6 | 0.54 | −0.13 | 0.96 | | |
| 19 | TRINITY_DN6 | 0.48 | −0.11 | 0.99 | | |
| 20 | comp22899_c | 0.4 | −0.19 | 0.91 | | |

# K−Means Cluster

## Spot Summary: B

# metagenes = 48
# genes = 40

<r> metagenes = 0.85
<r> genes = 0.77
beta: r2= 0.15 / log p= −1.22

# samples with spot = 0 ( 0 %)

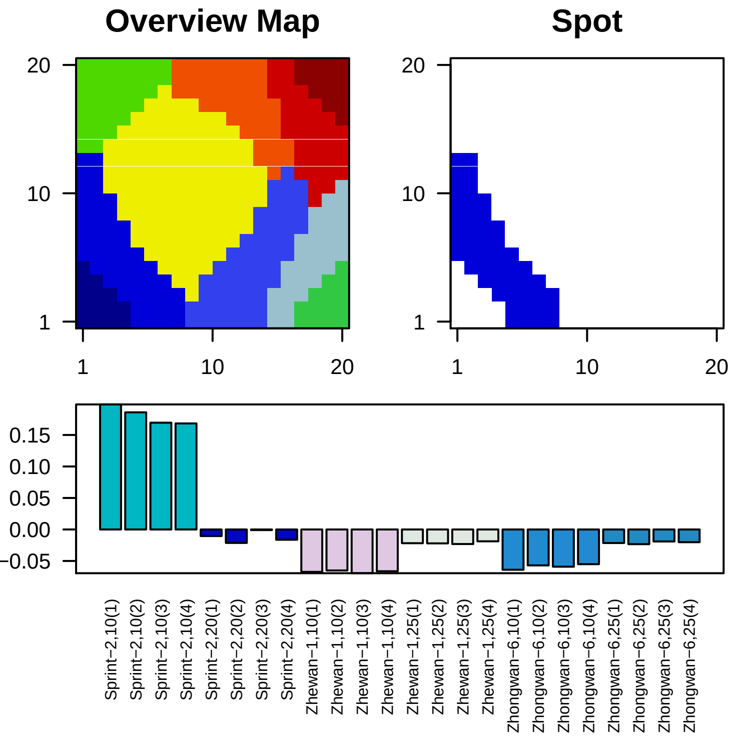


## Spot Genelist

| Rank | ID | max e | min e | r | Symbol | Description |
|---|---|---|---|---|---|---|
| 1 | TRINITY_DN1 | 0.55 | −0.07 | 0.9 | | |
| 2 | TRINITY_DN4 | 0.39 | −0.15 | 0.82 | | |
| 3 | TRINITY_DN1 | 0.39 | −0.07 | 0.97 | | |
| 4 | TRINITY_DN4 | 0.38 | −0.11 | 0.93 | | |
| 5 | TRINITY_DN7 | 0.37 | −0.08 | 0.97 | | |
| 6 | TRINITY_DN5 | 0.37 | −0.15 | 0.86 | | |
| 7 | TRINITY_DN3 | 0.36 | −0.05 | 0.89 | | |
| 8 | TRINITY_DN5 | 0.34 | −0.34 | 0.55 | | |
| 9 | TRINITY_DN1 | 0.33 | −0.25 | 0.86 | | |
| 10 | TRINITY_DN2 | 0.3 | −0.07 | 0.95 | | |
| 11 | TRINITY_DN6 | 0.29 | −0.24 | 0.85 | | |
| 12 | TRINITY_DN7 | 0.28 | −0.17 | 0.94 | | |
| 13 | TRINITY_DN4 | 0.26 | −0.04 | 0.94 | | |
| 14 | TRINITY_DN1 | 0.24 | −0.09 | 0.92 | | |
| 15 | TRINITY_DN7 | 0.24 | −0.13 | 0.96 | | |
| 16 | TRINITY_DN7 | 0.23 | −0.08 | 0.94 | | |
| 17 | TRINITY_DN7 | 0.23 | −0.22 | 0.67 | | |
| 18 | TRINITY_DN1 | 0.22 | −0.03 | 0.94 | | |
| 19 | TRINITY_DN8 | 0.22 | −0.12 | 0.96 | | |
| 20 | TRINITY_DN4 | 0.21 | −0.13 | 0.62 | | |

# K−Means Cluster

## Spot Summary: C

# metagenes = 31
# genes = 13

<r> metagenes = 0.97
<r> genes = 0.65
beta: r2= 0.63 / log p= −5.47

# samples with spot = 0 ( 0 %)

## Spot Genelist

| Rank | ID | max e | min e | r | Symbol | Description |
|---|---|---|---|---|---|---|
| 1 | TRINITY_DN4 | 1.16 | −0.51 | 0.71 | | |
| 2 | TRINITY_DN6 | 1.09 | −0.33 | 0.89 | | |
| 3 | comp40971_c | 0.9 | −0.39 | 0.64 | | |
| 4 | TRINITY_DN1 | 0.83 | −0.22 | 0.87 | | |
| 5 | TRINITY_DN6 | 0.82 | −0.22 | 0.87 | | |
| 6 | TRINITY_DN1 | 0.81 | −0.23 | 0.89 | | |
| 7 | TRINITY_DN4 | 0.7 | −0.29 | 0.94 | | |
| 8 | TRINITY_DN3 | 0.62 | −0.3 | 0.95 | | |
| 9 | TRINITY_DN1 | 0.61 | −0.36 | 0.83 | | |
| 10 | TRINITY_DN7 | 0.33 | −0.18 | 0.96 | | |
| 11 | TRINITY_DN3 | 0.31 | −0.25 | 0.32 | | |
| 12 | TRINITY_DN6 | 0.29 | −0.29 | 0.69 | | |
| 13 | TRINITY_DN4 | 0.28 | −0.24 | 0.77 | | |

**Overview Map**

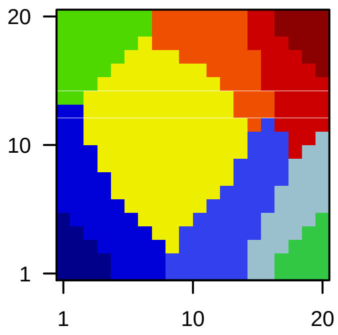


**Spot**

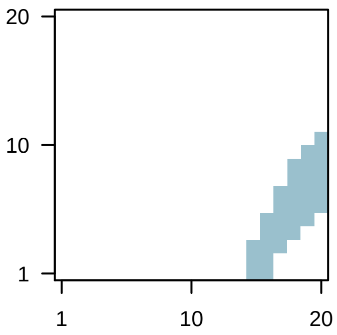


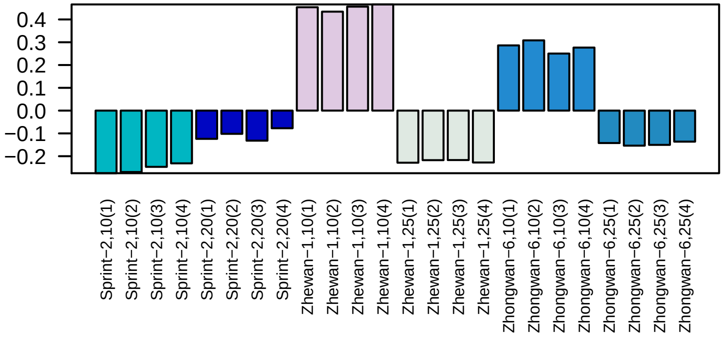

# K−Means Cluster

## Spot Summary: D

# metagenes = 14
# genes = 10

<r> metagenes = 1
<r> genes = 0.48
beta: r2= 4.67 / log p= −Inf

# samples with spot = 16 ( 66.7 %)
Sprint−2(20) : 4 ( 100 %)
Zhewan−1(10) : 4 ( 100 %)
Zhongwan−6(10) : 4 ( 100 %)
Zhongwan−6(25) : 4 ( 100 %)

**Overview Map**

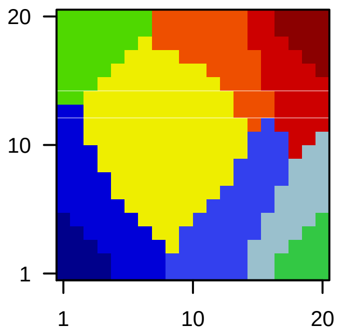


**Spot**

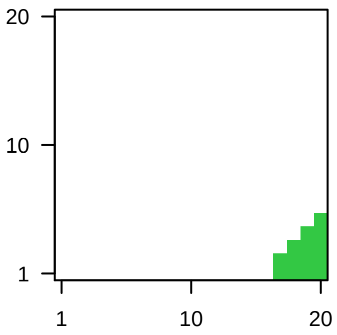


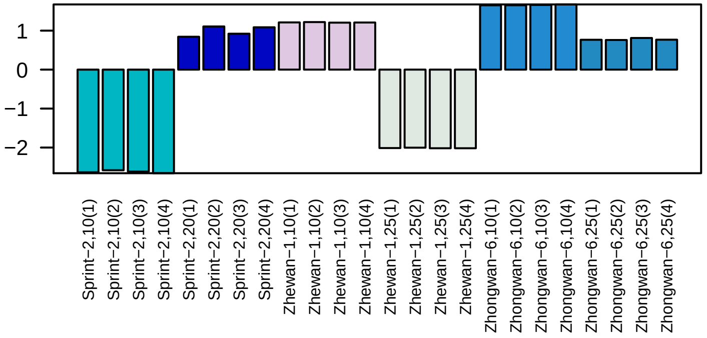


## Spot Genelist

| Rank | ID | max e | min e | r | Symbol | Description |
|---|---|---|---|---|---|---|
| 1 | TRINITY_DN1 | 9.22 | −1.86 | 0.58 | | |
| 2 | TRINITY_DN3 | 8.41 | −2.58 | 0.38 | | |
| 3 | TRINITY_DN1 | 5.71 | −13.31 | 0.99 | | |
| 4 | TRINITY_DN5 | 3.72 | −1.76 | 0.48 | | |
| 5 | TRINITY_DN2 | 2.73 | −3.16 | 0.83 | | |
| 6 | TRINITY_DN4 | 1.96 | −0.45 | 0.49 | | |
| 7 | TRINITY_DN2 | 1.83 | −1.59 | 0.53 | | |
| 8 | TRINITY_DN3 | 1.75 | −1.32 | 0.83 | | |
| 9 | TRINITY_DN6 | 1.68 | −1.13 | 0.6 | | |
| 10 | TRINITY_DN3 | 0.64 | −0.6 | 0.94 | | |

# K−Means Cluster

## Spot Summary: E

# metagenes = 34
# genes = 27

<r> metagenes = 0.94
<r> genes = 0.74
beta: r2= 0.39 / log p= −2.95

# samples with spot = 4 ( 16.7 %)
Zhewan−1(25) : 4 ( 100 %)

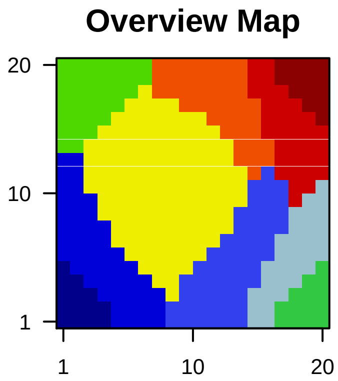


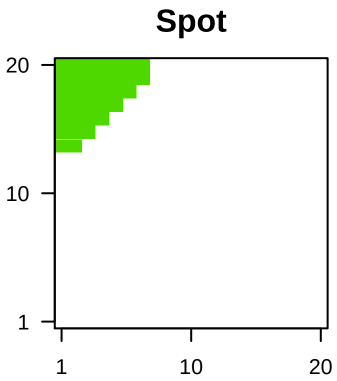


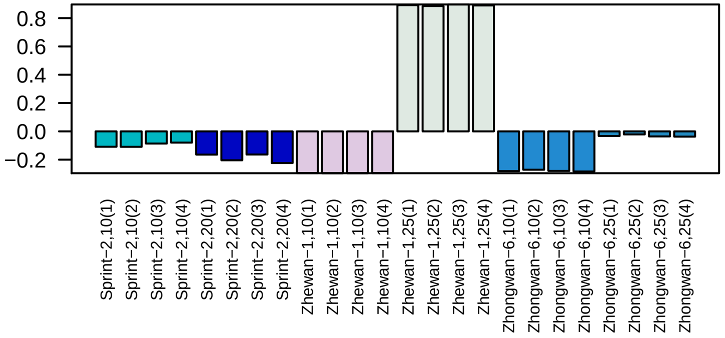


## Spot Genelist

| Rank | ID | max e | min e | r | Symbol | Description |
|---|---|---|---|---|---|---|
| 1 | TRINITY_DN3 | 15.54 | −3.43 | 0.98 | | |
| 2 | TRINITY_DN1 | 1.68 | −0.51 | 0.99 | | |
| 3 | TRINITY_DN1 | 0.79 | −0.37 | 0.93 | | |
| 4 | TRINITY_DN5 | 0.75 | −0.46 | 0.69 | | |
| 5 | TRINITY_DN5 | 0.74 | −0.39 | 0.91 | | |
| 6 | TRINITY_DN4 | 0.59 | −0.48 | 0.83 | | |
| 7 | TRINITY_DN1 | 0.52 | −0.35 | 0.77 | | |
| 8 | TRINITY_DN5 | 0.47 | −0.16 | 0.97 | | |
| 9 | TRINITY_DN1 | 0.4 | −0.22 | 0.86 | | |
| 10 | TRINITY_DN6 | 0.37 | −0.25 | 0.64 | | |
| 11 | TRINITY_DN3 | 0.29 | −0.14 | 0.77 | | |
| 12 | TRINITY_DN6 | 0.28 | −0.1 | 0.94 | | |
| 13 | TRINITY_DN3 | 0.26 | −0.26 | 0.45 | | |
| 14 | TRINITY_DN1 | 0.22 | −0.06 | 0.99 | | |
| 15 | TRINITY_DN4 | 0.22 | −0.09 | 0.91 | | |
| 16 | TRINITY_DN5 | 0.21 | −0.28 | 0.64 | | |
| 17 | TRINITY_DN1 | 0.2 | −0.07 | 0.98 | | |
| 18 | TRINITY_DN3 | 0.2 | −0.1 | 0.88 | | |
| 19 | TRINITY_DN1 | 0.2 | −0.06 | 0.98 | | |
| 20 | TRINITY_DN1 | 0.19 | −0.11 | 0.93 | | |

# K−Means Cluster

## Spot Summary: F

# metagenes = 118
# genes = 445

<r> metagenes = 0.01
<r> genes = 0.02
beta: r2= 0 / log p= −0.03

# samples with spot = 0 ( 0 %)

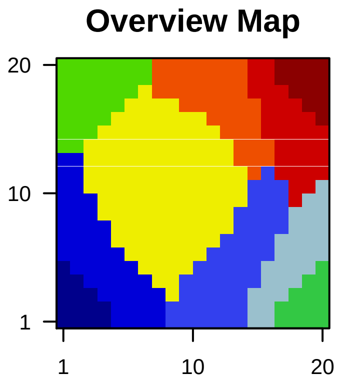


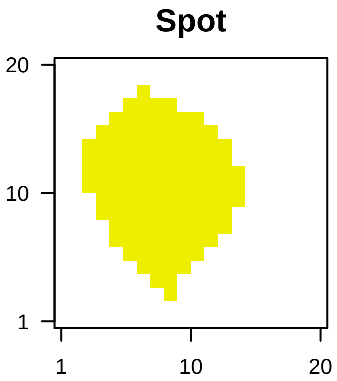


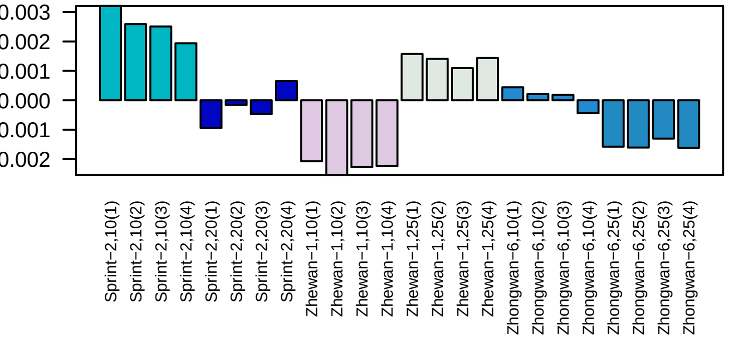


## Spot Genelist

| Rank | ID | max e | min e | r | Symbol | Description |
|---|---|---|---|---|---|---|
| 1 | TRINITY_DN5 | 0.27 | −0.05 | −0.41 | | |
| 2 | TRINITY_DN6 | 0.27 | −0.09 | −0.05 | | |
| 3 | comp39525_c | 0.2 | −0.08 | 0.22 | | |
| 4 | TRINITY_DN6 | 0.2 | −0.05 | 0.32 | | |
| 5 | TRINITY_DN5 | 0.2 | −0.03 | −0.6 | | |
| 6 | TRINITY_DN9 | 0.19 | −0.06 | 0.1 | | |
| 7 | TRINITY_DN4 | 0.18 | −0.11 | 0.1 | | |
| 8 | TRINITY_DN3 | 0.18 | −0.03 | −0.06 | | |
| 9 | TRINITY_DN2 | 0.17 | −0.03 | 0.02 | | |
| 10 | comp69498_c | 0.17 | −0.02 | 0.06 | | |
| 11 | TRINITY_DN8 | 0.16 | −0.14 | 0.18 | | |
| 12 | TRINITY_DN1 | 0.15 | −0.1 | −0.16 | | |
| 13 | TRINITY_DN1 | 0.15 | −0.05 | 0.04 | | |
| 14 | TRINITY_DN1 | 0.15 | −0.05 | −0.18 | | |
| 15 | TRINITY_DN4 | 0.15 | −0.13 | −0.19 | | |
| 16 | TRINITY_DN9 | 0.15 | −0.03 | −0.4 | | |
| 17 | TRINITY_DN2 | 0.14 | −0.02 | 0 | | |
| 18 | TRINITY_DN3 | 0.14 | −0.08 | 0.75 | | |
| 19 | TRINITY_DN1 | 0.13 | −0.02 | −0.11 | | |
| 20 | TRINITY_DN1 | 0.13 | −0.06 | 0.44 | | |

# K−Means Cluster

## Spot Summary: G

# metagenes = 41
# genes = 19

<r> metagenes = 0.82
<r> genes = 0.5
beta: r2= 0.09 / log p= −0.82

# samples with spot = 0 ( 0 %)

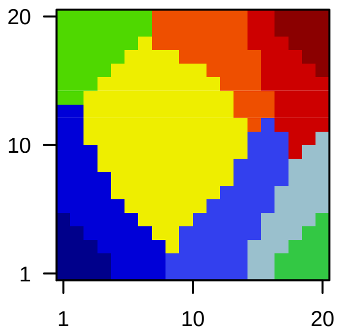


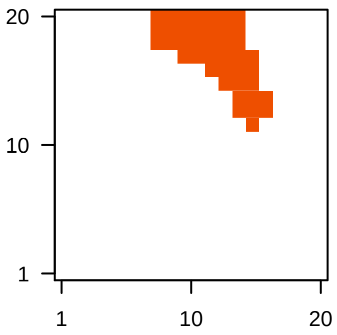


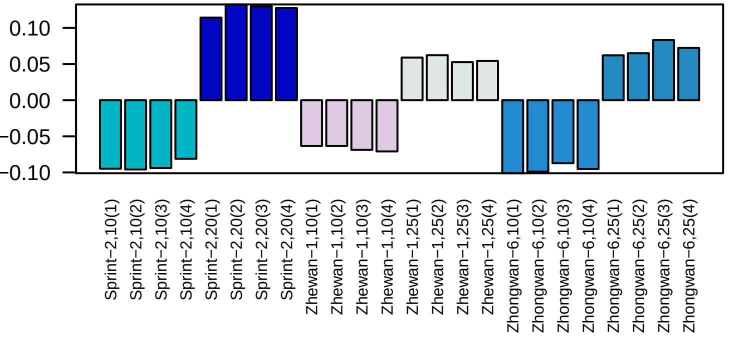


## Spot Genelist

| Rank | ID | max e | min e | r | Symbol | Description |
|---|---|---|---|---|---|---|
| 1 | comp33849_c | 1.11 | −0.57 | 0.76 | | |
| 2 | TRINITY_DN6 | 0.38 | −0.27 | 0.93 | | |
| 3 | TRINITY_DN5 | 0.35 | −0.26 | 0.88 | | |
| 4 | TRINITY_DN9 | 0.35 | −0.08 | 0.39 | | |
| 5 | TRINITY_DN4 | 0.31 | −0.06 | 0.69 | | |
| 6 | TRINITY_DN4 | 0.29 | −0.27 | 0.94 | | |
| 7 | TRINITY_DN4 | 0.28 | −0.15 | 0.84 | | |
| 8 | TRINITY_DN2 | 0.26 | −0.12 | 0.78 | | |
| 9 | TRINITY_DN5 | 0.16 | −0.09 | 0.42 | | |
| 10 | TRINITY_DN3 | 0.15 | −0.08 | 0.96 | | |
| 11 | TRINITY_DN3 | 0.14 | −0.08 | 0.18 | | |
| 12 | TRINITY_DN8 | 0.14 | −0.14 | 0.9 | | |
| 13 | TRINITY_DN6 | 0.12 | −0.08 | 0.69 | | |
| 14 | TRINITY_DN2 | 0.1 | −0.09 | 0.41 | | |
| 15 | TRINITY_DN1 | 0.1 | −0.08 | 0.78 | | |
| 16 | TRINITY_DN1 | 0.09 | −0.09 | 0.55 | | |
| 17 | TRINITY_DN5 | 0.08 | −0.05 | 0.5 | | |
| 18 | TRINITY_DN2 | 0.06 | −0.05 | 0.87 | | |
| 19 | TRINITY_DN4 | 0.05 | −0.12 | 0.43 | | |

# K−Means Cluster

## Spot Summary: H

# metagenes = 52
# genes = 45

<r> metagenes = 0.82
<r> genes = 0.5
beta: r2= 0.15 / log p= −1.23

# samples with spot = 0 ( 0 %)

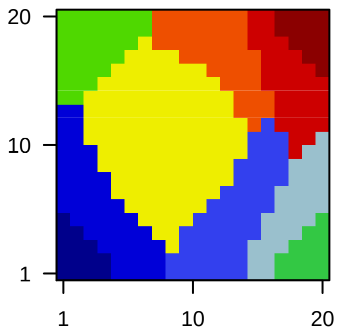


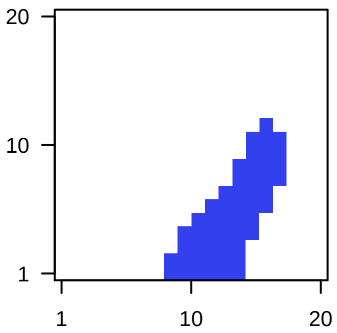


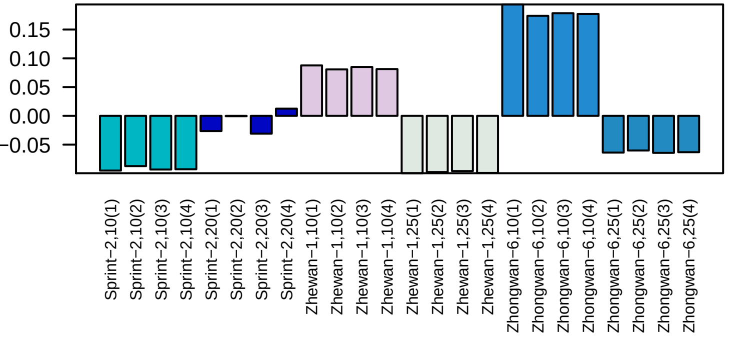


## Spot Genelist

| Rank | ID | max e | min e | r | Symbol | Description |
|---|---|---|---|---|---|---|
| 1 | TRINITY_DN1 | 1.04 | −0.18 | 0.77 | | |
| 2 | TRINITY_DN1 | 1.03 | −0.19 | 0.8 | | |
| 3 | TRINITY_DN2 | 1 | −0.17 | 0.8 | | |
| 4 | TRINITY_DN4 | 0.62 | −0.35 | 0.19 | | |
| 5 | TRINITY_DN1 | 0.6 | −0.15 | 0.92 | | |
| 6 | TRINITY_DN3 | 0.57 | −0.12 | 0.87 | | |
| 7 | TRINITY_DN2 | 0.57 | −0.1 | 0.82 | | |
| 8 | TRINITY_DN3 | 0.54 | −0.17 | 0.49 | | |
| 9 | TRINITY_DN2 | 0.53 | −0.16 | 0.58 | | |
| 10 | TRINITY_DN1 | 0.53 | −0.11 | 0.85 | | |
| 11 | TRINITY_DN3 | 0.52 | −0.14 | 0.88 | | |
| 12 | TRINITY_DN3 | 0.47 | −0.23 | 0.96 | | |
| 13 | TRINITY_DN5 | 0.44 | −0.12 | 0.43 | | |
| 14 | TRINITY_DN1 | 0.42 | −0.18 | 0.95 | | |
| 15 | TRINITY_DN4 | 0.42 | −0.06 | 0.78 | | |
| 16 | TRINITY_DN3 | 0.36 | −0.11 | 0.26 | | |
| 17 | TRINITY_DN2 | 0.34 | −0.07 | 0.91 | | |
| 18 | TRINITY_DN5 | 0.32 | −0.18 | 0.89 | | |
| 19 | TRINITY_DN4 | 0.31 | −0.18 | 0.38 | | |
| 20 | TRINITY_DN1 | 0.3 | −0.22 | 0.62 | | |

# K−Means Cluster

## Spot Summary: I

# metagenes = 34
# genes = 4

<r> metagenes = 0.94
<r> genes = 0.75
beta: r2= 0.62 / log p= −5.29

# samples with spot = 0 ( 0 %)

## Spot Genelist

| Rank | ID | max e | min e | r | Symbol | Description |
|---|---|---|---|---|---|---|
| 1 | TRINITY_DN4 | 0.63 | −0.35 | 0.97 | | |
| 2 | comp15249_c | 0.39 | −0.2 | 0.85 | | |
| 3 | TRINITY_DN5 | 0.29 | −0.43 | 0.79 | | |
| 4 | TRINITY_DN1 | 0.27 | −0.18 | 0.84 | | |

**Overview Map**

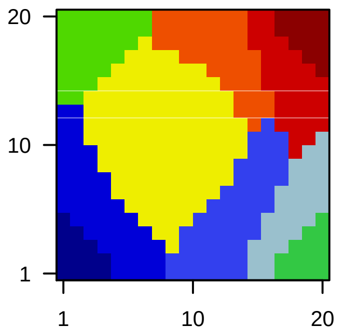


**Spot**

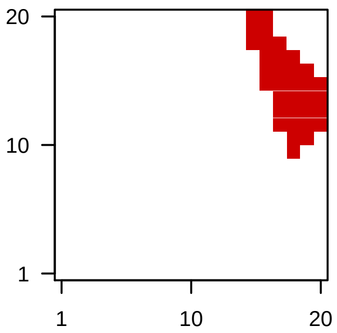


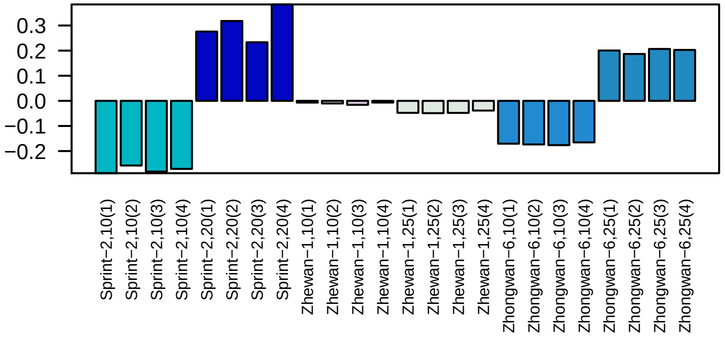

# K−Means Cluster

## Spot Summary: J

# metagenes = 14
# genes = 8

<r> metagenes = 0.99
<r> genes = 0.55
beta: r2= 2.54 / log p= −Inf

# samples with spot = 8 ( 33.3 %)
Zhewan−1(25) : 4 ( 100 %)
Zhongwan−6(25) : 4 ( 100 %)

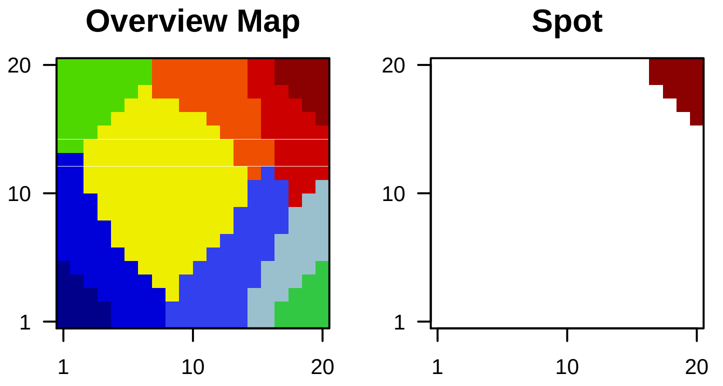


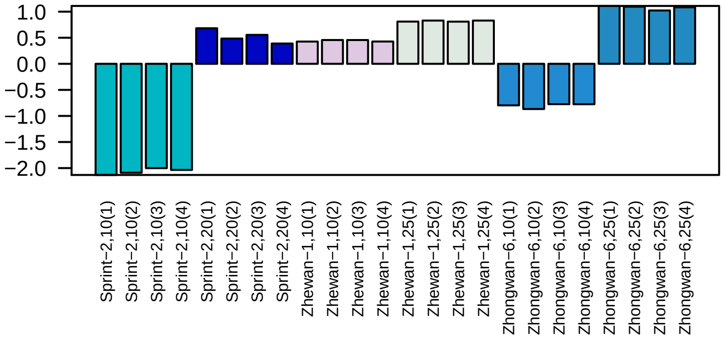


## Spot Genelist

| Rank | ID | max e | min e | r | Symbol | Description |
|---|---|---|---|---|---|---|
| 1 | TRINITY_DN1 | 3.01 | −8.1 | 0.98 | | |
| 2 | TRINITY_DN3 | 2.24 | −1.85 | 0.77 | | |
| 3 | TRINITY_DN4 | 1.68 | −0.51 | 0.56 | | |
| 4 | TRINITY_DN6 | 1.58 | −2.5 | 0.89 | | |
| 5 | TRINITY_DN2 | 1.45 | −0.95 | 0.56 | | |
| 6 | TRINITY_DN7 | 1.1 | −1.67 | 0.67 | | |
| 7 | TRINITY_DN6 | 0.64 | −0.76 | 0.85 | | |
| 8 | TRINITY_DN3 | 0.57 | −0.79 | 0.58 | | |
